# A high-quality chromosome-level wild rice genome of *Oryza coarctata*

**DOI:** 10.1038/s41597-023-02594-1

**Published:** 2023-10-14

**Authors:** Hang Zhao, Wenzheng Wang, Yirong Yang, Zhiwei Wang, Jing Sun, Kaijun Yuan, S. M. Hisam Al Rabbi, Munnujan Khanam, Md. Shahjahan Kabir, Zeba I. Seraj, Md. Sazzadur Rahman, Zhiguo Zhang

**Affiliations:** 1 Biotechnology Research Institute, Chinese Academy of Agricultural Sciences, Beijing, 100081 China; 2 Gembloux Agro-Bio Tech, TERRA Teaching and Research Centre, University of Liège, Gembloux, Belgium; 3 Bangladesh Rice Research Institute, Gazipur, 1701 Bangladesh; 4 Department of Biochemistry and Molecular Biology, University of Dhaka, Dhaka, Bangladesh; 5 Present Address: Duke university, Durham, USA

**Keywords:** Plant evolution, Next-generation sequencing

## Abstract

*Oryza coarctata* (2n = 4X = 48, KKLL) is an allotetraploid, undomesticated relative of rice and the only species in the genus *Oryza* with tolerance to high salinity and submergence. Therefore, it contains important stress and tolerance genes/factors for rice. The initial draft genome published was limited by data and technical restrictions, leading to an incomplete and highly fragmented assembly. This study reports a new, highly contiguous chromosome-level genome assembly and annotation of *O. coarctata*. PacBio high-quality HiFi reads generated 460 contigs with a total length of 573.4 Mb and an N50 of 23.1 Mb, which were assembled into scaffolds with Hi-C data, anchoring 96.99% of the assembly onto 24 chromosomes. The genome assembly comprises 45,571 genes, and repetitive content contributes 25.5% of the genome. This study provides the novel identification of the KK and LL genome types of the genus *Oryza*, leading to valuable insights into rice genome evolution. The chromosome-level genome assembly of *O. coarctata* is a valuable resource for rice research and molecular breeding.

## Background & Summary

**Fig. 1:**
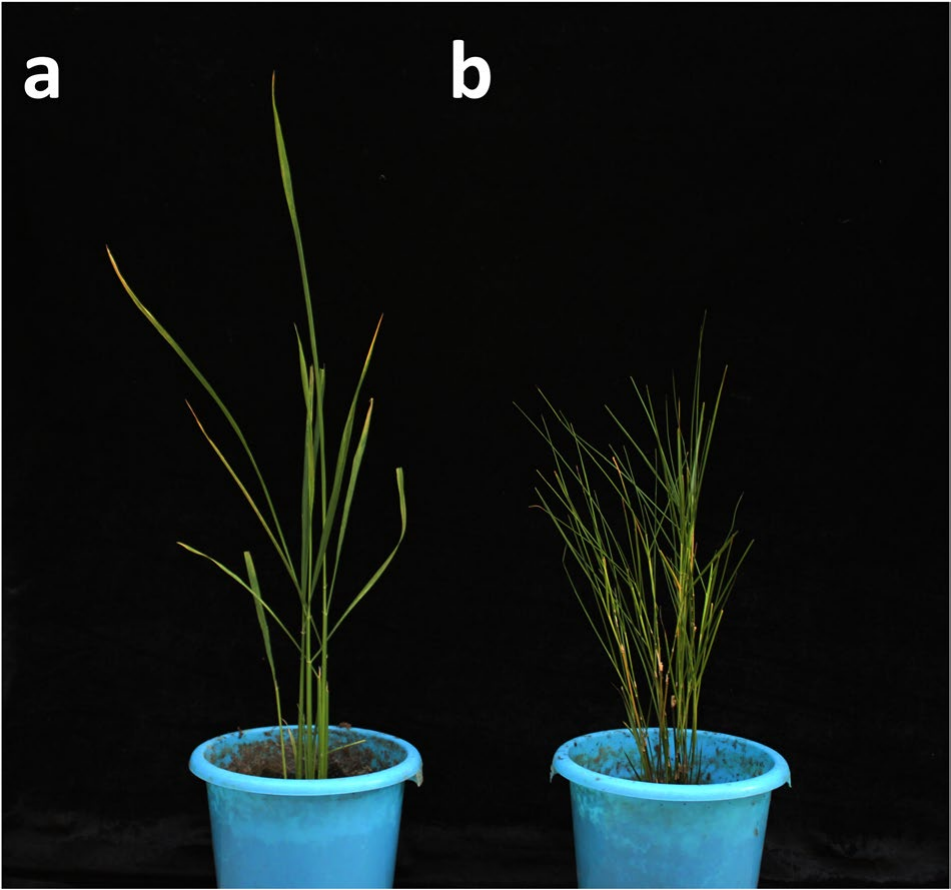
Photographs of rice plants. (**a**) *Oryza sativa*. (**b**) *Oryza coarctata*.

**Fig. 2:**
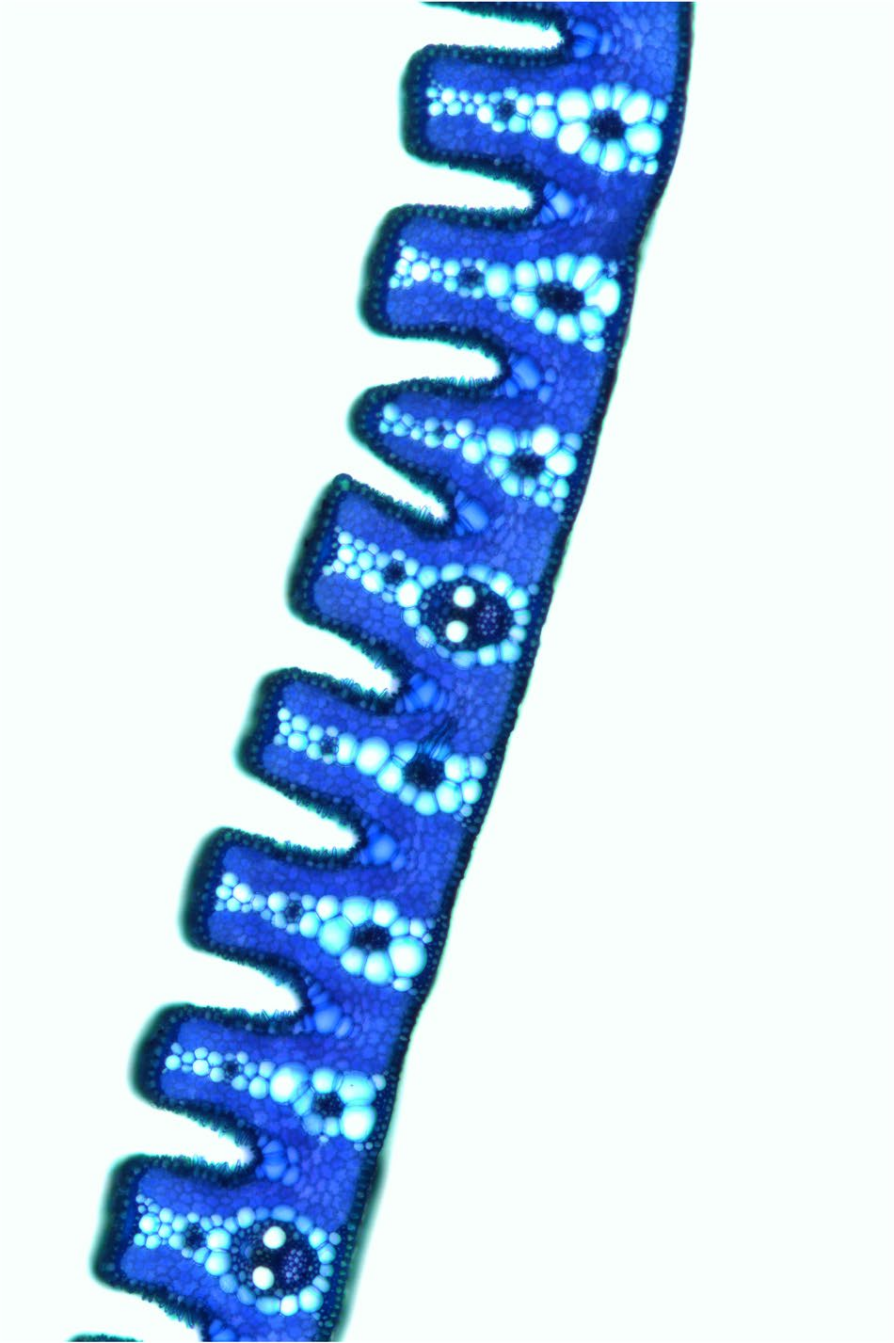
Transverse section of young leaf of *O. coarctata* under light microscope.

*Oryza coarctata* is the only halophyte species in the genus *Oryza*^1^, exhibiting distinct natural traits, including high tolerance to submersion and salinity^2^ (Fig. 1), as well as unique leaf anatomical features, such as the presence of Kranz anatomy (Fig. 2). These features are a result of high selection pressure, allowing its propagation in a wide range of ecological conditions, from submerged saline to non-saline terrestrial soils^3^. *Oryza coarctata* is primarily found in coastal areas across Bangladesh, India, Sri Lanka, and Malaysia^1,2,4,5^.

The history of research on *Oryza coarctata* is complex. Until 1999, it was excluded from the rice genus due to some morphological differences and was classified as *Porteresia coarctata*. However, a study on the evolutionary relationship between various species and genome types of the rice genus demonstrated that it belongs to the genus *Oryza*, and it was named *Oryza coarctata*. It was also determined to be allotetraploid^6^. A subsequent study identified the genome types, KK and LL, from its allotetraploid genome^7^.

Numerous studies have demonstrated that *Oryza coarctata* offers a wealth of genetic resources for rice breeding research, including salt resistance, drought resistance, and improved photosynthetic efficiency^2,3,8,9^. Therefore, sequencing a high-quality chromosome-level genome of *Oryza coarctata* is essential for genomics research and can provide new insights into the evolutionary studies of rice. In our study, we sequenced a high-quality chromosome-level genome of *Oryza coarctata* using PacBio HiFi reads (~59.99 X) and Hi-C data. We also identified the genome types, KK and LL, from its allotetraploid genome, which can provide new insights into the evolution of genus *Oryza*.

## Methods

### Staining method of *O. coarctata* leaf

Fresh leaf samples from three-leaf-stage plants were selected and snipped into 1cm-by-1cm squares. Immediately, these samples were placed in Carnoy’s fixative (a mixture of ethanol and acetic acid in a 3:1 ratio). After being fixed at room temperature for 48 hours, the samples were shifted to 75% ethanol for permanent preservation. If proceeding with subsequent experiments, slices were manually prepared using a double blade, perpendicular to the leaf veins. The prepared filamentous sections were stained using 0.1% methylene blue for 3 minutes. Once stained, excess dye was rinsed off, and the samples were set on microscope slides for observation under a light microscope.

### Genome sequencing

We began with fresh *Oryza coarctata* seedlings, sourced from the Koyra Riverbank in Khulna district, Bangladesh (22.77 N latitude and 89.48 E longitude), and utilized them for superior DNA extraction. Our extraction protocol involved initial fragmentation of DNA samples via a g-TUBE, subsequent repair of damaged DNA, end repair, and ligation with dumbbell-shaped adapters. After an exonuclease digestion, we screened the DNA fragments using BluePippin, forming the PacBio sequencing library. For the Hi-C library, we employed formaldehyde for crosslinking cells, thereby maintaining both intra- and intermolecular interactions, and preserving the cell’s 3D structure. Following crosslinking, we employed the restriction enzyme HindIII for DNA digestion and incorporated biotin-labeled nucleotides during the end repair stage. After ligation of the repaired ends, we circularized the DNA, which enabled the identification of interactive DNA positions in further sequencing and analyses. We then decrosslinked and purified the DNA, fragmenting it into 300–700 bp lengths. Interaction-representing biotin-labeled DNA fragments were captured with streptavidin magnetic beads, thereby facilitating library construction. We sequenced the PacBio library on the PacBio Sequel II system (CCS mode), generating ~34.4 Gb clean data (~59.99 × ), and all the CCS reads exhibited an N50 of ~15.2 kb. The Hi-C library, sequenced on the Illmina NovaSeq 6000 (PE150), produced ~73.76 Gb clean data (Table 1).

**Table 1:** Sequencing data for *Oryza coarctata* genome assembly.

Sequencing Strategy	Sequencing Platform	Reads Number	Clean Data (Gb)	Sequence Coverage (X)
PacBio	PacBio Sequel II	2,295,034	34.40	59.99
Hi-C	Illumina NovaSeq 6000 PE150	246,915,123	73.76	128.63
RNA	Illumina NovaSeq 6000 PE150	44,284,836	13.23	23.09

Note that the sequencing coverage is calculated by the genome size of 573Mb.

### RNA sequencing

RNA was extracted from the root, stem, and leaf tissues of *Oryza coarctata* plants. Following extraction, these RNA samples were combined in equal measures, from which an RNA-seq library was prepared. The transcriptomes were sequenced on the Illmina NovaSeq. 6000 platform, operated by the Biomarker Technology Company, Beijing, China. The sequencing process produced 13.23 Gb of short-read RNA-seq data (Table 1), which was used for predicting whole-genome protein-coding genes.

### Genome assembly

**Fig. 3:**
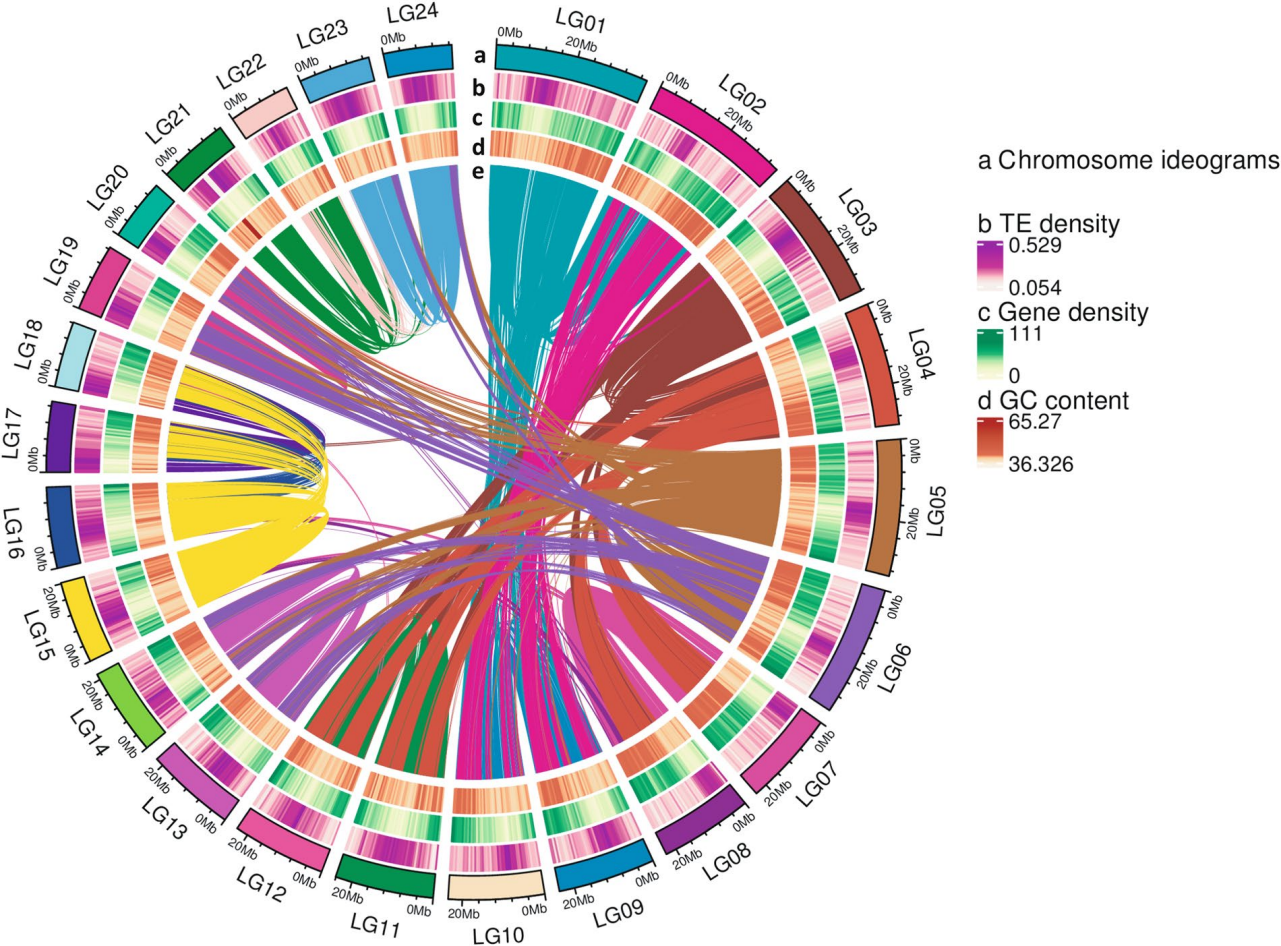
Genome features of the *O. coarctata*. (**a**) Chromosome ideograms of *O. coarctata* genome. (**b**) TE density. (**c**) Gene density. (**d**) GC content. (**e**) Syntenic blocks of genome sequence.

**Fig. 4:**
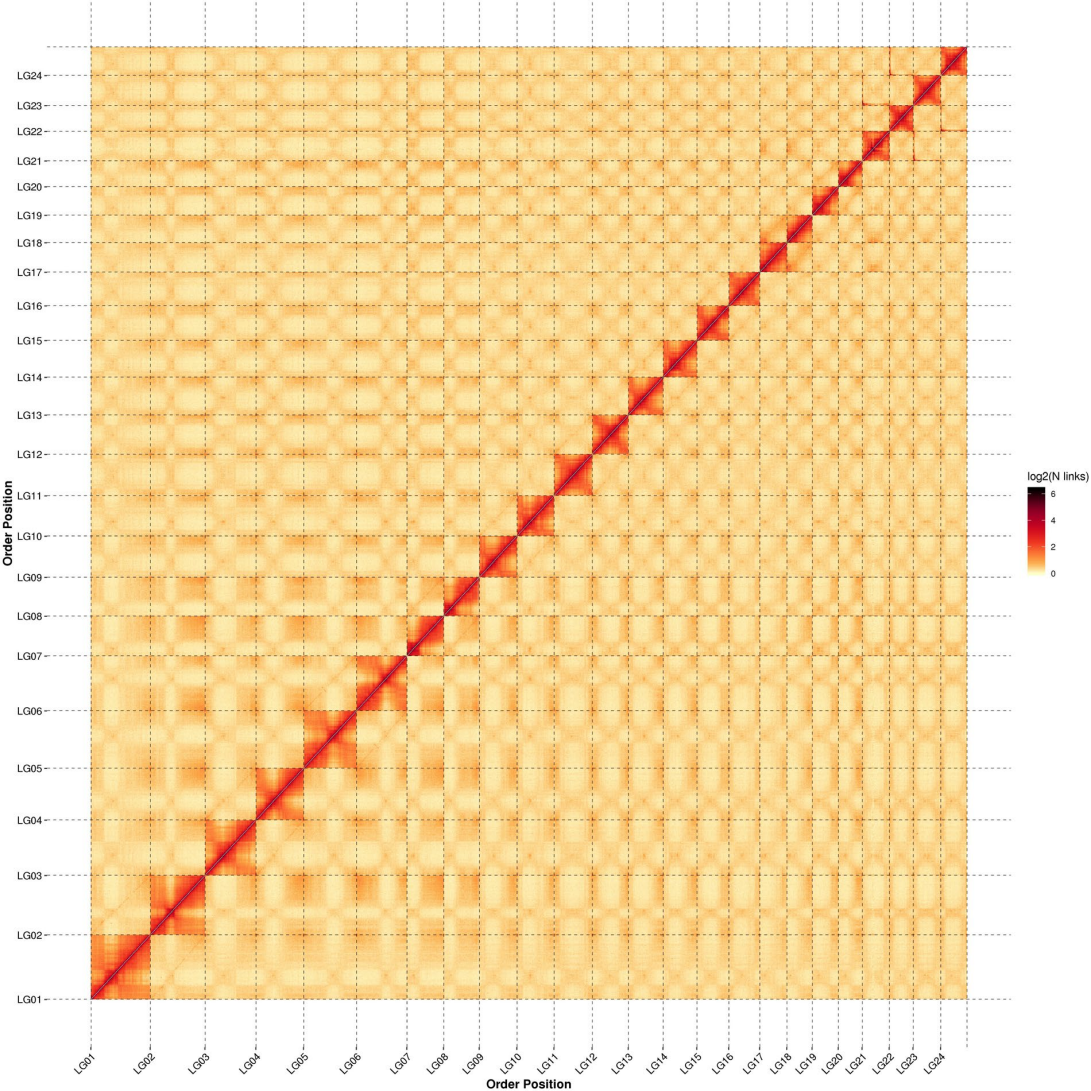
Hi-C contact map of the chromosome-level assembly of *O. coarctata*. The intensity of interactions was calculated using a bin size of 300,000 bp.

We used hifiasm software^10^ to assemble the high-quality HiFi reads, which yielded a total of 460 contigs with a total length of 573.4 Mb and an N50 of 23.1 Mb (Table 2). Using Hi-C data, more than 96% of the contigs have been anchor to 24 chromosomes (Fig. 3). Subsequently, we joined contigs into scaffolds using Hi-C clean data. The 46.55% of unique mapped read pairs were valid interaction pairs and were used for correction of scaffolds and clustered, ordered and orientated scaffolds onto chromosomes by LACHESIS^11^. Before the assembly of chromosomes, we first executed a preassembly phase to correct errors in scaffolds, requiring the division of scaffolds into average segments of 50 kb. The Hi-C data were then mapped to these segments using the BWA (version 0.7.10-r789)^12^ software. We preserved uniquely mapped data for assembly operations using LACHESIS software. We manually checked any pair of segments that exhibited inconsistent connection with the raw scaffold data. These corrected scaffolds were subsequently assembled using LACHESIS. After this process, we manually adjusted any placement and orientation errors that exhibited distinct chromatin interaction patterns. In the end, we anchored 24 scaffolds, amounting to 96.99% total length, to the chromosomes (Fig. 4, Table 3).

**Table 2:** The Assembly Results for *Oryza coarctata* genome assembly.

Contig Number	Contig Length (bp)	Contig N50 (bp)	Contig N90 (bp)	Contig Max length (bp)	GC(%)
460	573,362,877	23,112,565	16,161,634	37,520,647	42.06

**Table 3:** Statistics for Chromosome-level assembly of the *Oryza coarctata* genome.

Group	Cluster Number	Cluster Length	Order Number	Order Length
LG01	1	37,520,647	1	37520647
LG02	1	34,547,619	1	34547619
LG03	1	32,136,232	1	32136232
LG04	1	30,021,989	1	30021989
LG05	2	33,347,948	1	33312109
LG06	1	31,981,162	1	31981162
LG07	3	23,112,365	3	23112365
LG08	1	22,654,099	1	22654099
LG09	1	23,765,581	1	23765581
LG10	3	23,413,980	3	23413980
LG11	12	24,325,773	5	24110266
LG12	1	22,920,526	1	22920526
LG13	1	22,060,326	1	22060326
LG14	3	21,468,510	1	21392239
LG15	1	20,220,866	1	20220866
LG16	1	19,768,980	1	19768980
LG17	1	17,178,965	1	17178965
LG18	1	16,161,634	1	16161634
LG19	1	16,539,510	1	16539510
LG20	1	15,069,377	1	15069377
LG21	7	17,376,405	1	17196773
LG22	16	15,800,443	4	15125575
LG23	5	18,051,338	3	17496548
LG24	1	16,671,748	1	16671748
Total(Ratio %)	67(14.41)	556,116,023(96.99)	37(55.22)	554,379,116(99.69)

### Repeat annotation

Transposon element (TE) and tandem repeat were masked and annotated by the following workflows. TE were identified by a combination of homology-based and de novo approaches. We first customized a de novo repeat library of the genome using RepeatModeler^13^, which can automatically execute two de novo repeat finding programs, including RECON (version 1.08)^14^ and RepeatScout^15^.Then full-length long terminal repeat retrotransposons (fl-LTR-RTs) were identified using both LTRharvest^16^ and LTR_finder^17^. The high-quality intact fl-LTR-RTs and non-redundant LTR library were then produced by LTR_retriever^18^. Non-redundant species-specific TE library was constructed by combining the de novo TE sequences library above with the known Repbase (version 19.06)^19^, REXdb (V3.0)^20^ and Dfam (v3.2)^21^ database. Final TE sequences in the *Oryza coarctata* genome were identified and classified by homology search against the library using RepeatMasker v4.10^22^. Tandem repeats were annotated by Tandem Repeats Finder^23^ and MIcroSAtellite identification tool (MISA v2.1)^24^ (Tables 4, 5).

**Table 4:** Summary of the TE sequences in the *Oryza coarctata* genome.

Type	Number	Length (bp)	Rate(%)
ClassI:Retroelement	55,944	107,007,850	18.66
ClassI/DIRS	337	19,892	0
ClassI/LINE	4,547	6,782,099	1.18
ClassI/LTR/Cassandra	133	8,431	0
ClassI/LTR/Caulimovirus	193	356,873	0.06
ClassI/LTR/Copia	16,743	46,946,008	8.19
ClassI/LTR/ERV	2,226	156,930	0.03
ClassI/LTR/Gypsy	21,731	46,461,892	8.1
ClassI/LTR/Pao	312	33,233	0.01
ClassI/LTR/Unknown	8,474	6,092,128	1.06
ClassI/LTR/Viper	18	3,950	0
ClassI/SINE	1,230	146,414	0.03
ClassII:DNA transposon	58,309	24,238,025	4.23
ClassII/Academ	15	1,101	0
ClassII/CACTA	4,585	5,683,817	0.99
ClassII/Crypton	170	17,219	0
ClassII/Dada	104	96,756	0.02
ClassII/EnSpm	13	6,816	0
ClassII/Ginger	90	5,223	0
ClassII/Helitron	1,028	1,169,159	0.2
ClassII/IS3EU	57	3,681	0
ClassII/Kolobok	158	9,930	0
ClassII/MITE	23,738	5,163,657	0.9
ClassII/Maverick	210	12,093	0
ClassII/Merlin	14	3,505	0
ClassII/Mutator	3,979	5,350,285	0.93
ClassII/Novosib	112	6,944	0
ClassII/P	90	5,448	0
ClassII/PIF-Harbinger	2,484	677,588	0.12
ClassII/PiggyBac	206	16,411	0
ClassII/Sola	58	3,258	0
ClassII/Stowaway	8	576	0
ClassII/Tc1-Mariner	9,125	1,804,158	0.31
ClassII/Tourist	123	16,430	0
ClassII/Unknown	9,014	2,269,727	0.4
ClassII/Zator	9	607	0
ClassII/Zisupton	98	11,479	0
ClassII/hAT	2,821	1,902,157	0.33
Total	114,253	131,245,875	22.89

**Table 5:** Summary of the tandem repeat sequences in the *Oryza coarctata* genome.

Type	Number	Length	Rate(%)
Microsatellite (1–9 bp units)	100,902	2,514,980	0.44
Minisatellite (10–99 bp units)	1,611	1,626,981	0.28
Satellite (> = 100 bp units)	5,579	10,901,159	1.9
Total	108,092	15,043,120	2.62

### Gene prediction annotation of the genome

**Fig. 5:**
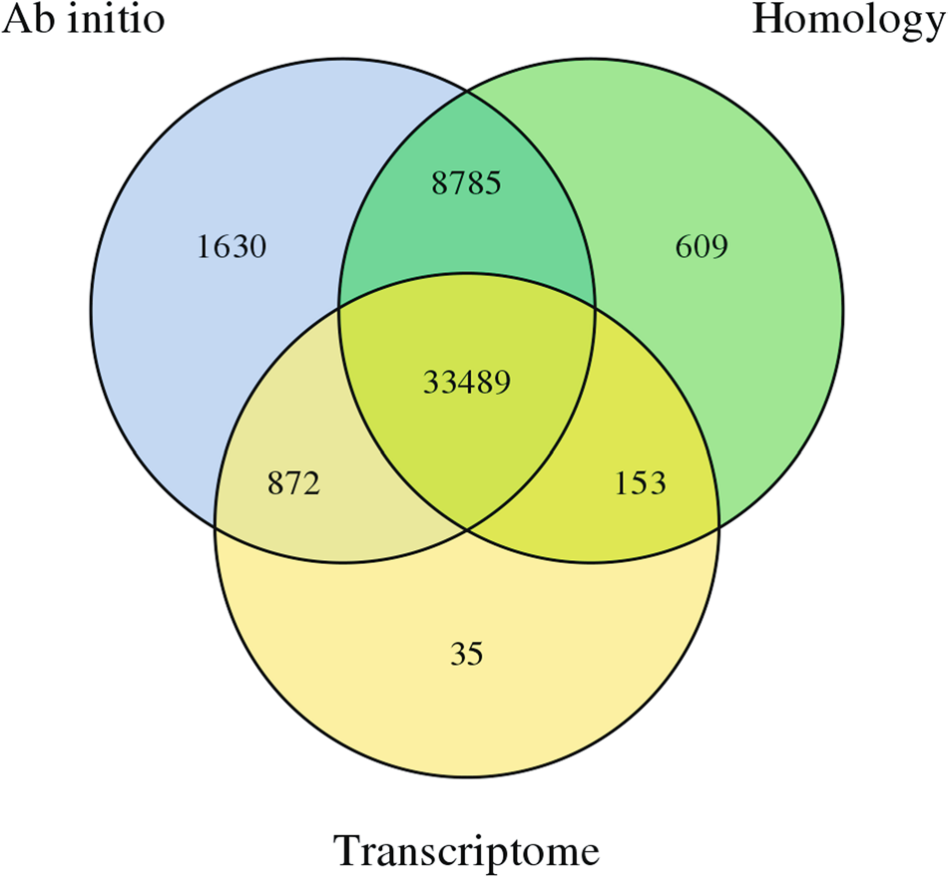
The genes that are integrated originated from the distribution maps of three prediction methods.

Gene prediction is typically performed using three methods: homology-based prediction, de novo prediction, and transcriptome-based prediction. De novo prediction was performed using Augustus v2.4^25^ and SNAP (2006-07-28)^26^. Homology-based prediction was performed using GeMoMa v1.7^27^ based on homologous species. Transcriptome-based prediction was conducted using both reference-based and de novo transcriptome assembly. Reference-based transcriptome assembly was performed using Hisat v2.0.4^28^ and Stringtie v1.2.3^29^, and GeneMarkS-T v5.1^30^ was used for gene prediction. De novo transcriptome assembly was performed using Trinity v2.11^31^, and gene prediction was conducted using PASA v2.0.2^32^. Finally, EVM v1.1.1^33^ was used to integrate the results from the three methods, and PASA v2.0.2 was used for annotation, resulting in 45,571 predicted genes (Fig. 5).

**Fig. 6:**
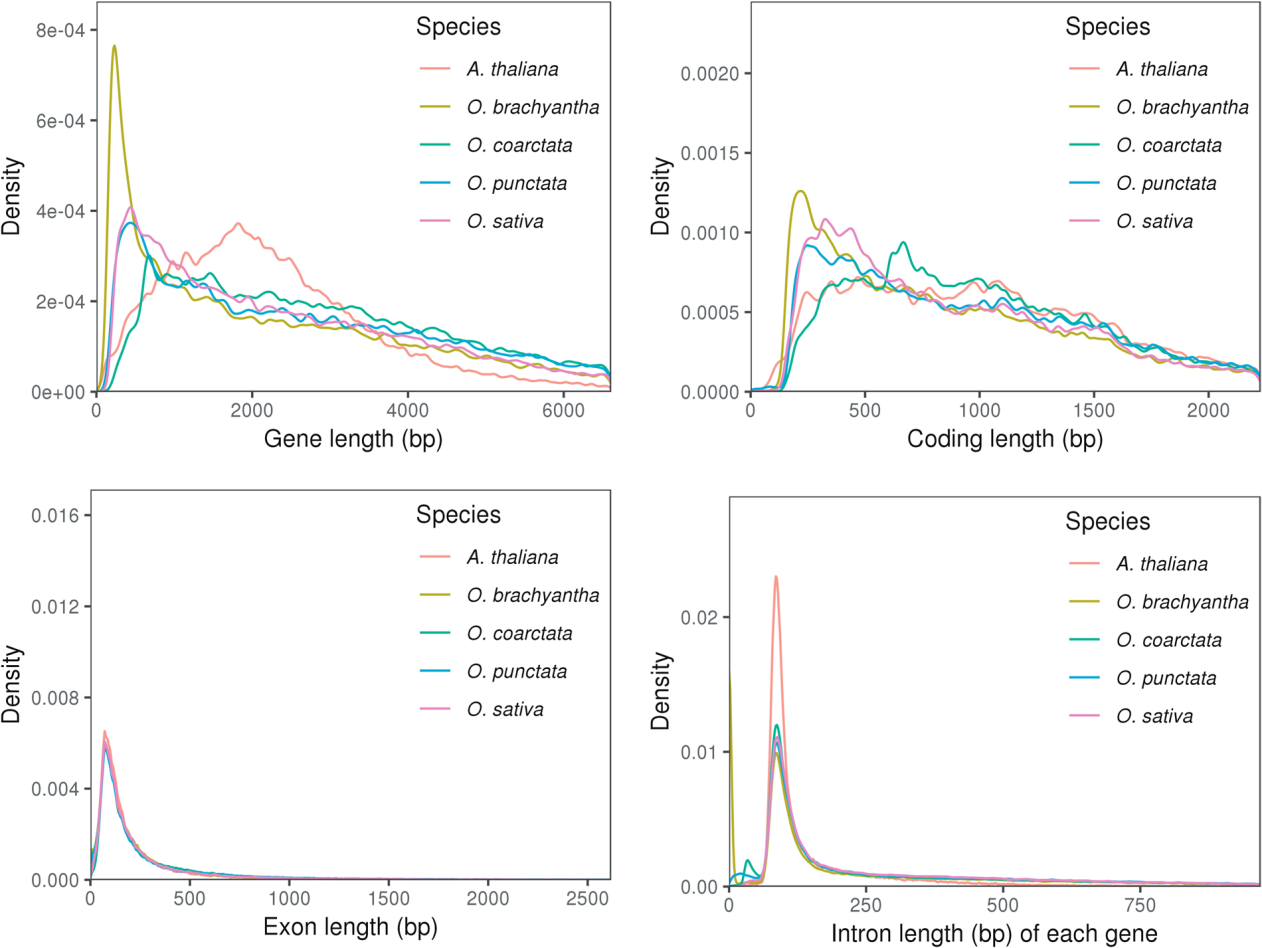
Comparisons of gene features among *O. coarctata* and the four other species (*A. thaliana*, *O. brachyantha*, *O. punctata* and *O. sativa*). Gene features include gene length, CDS length, exon length and intron length.

In order to evaluate the accuracy of gene prediction, we compared the length distributions of protein-coding genes, coding sequences (CDS), exons, and introns of our study species with those from four additional reference species (*A. thaliana*^34^, *O. brachyantha*^35^, *O. punctata*^36^, and *O. sativa*^37^). Notably, we did not observe any significant differences in the length distribution of gene features among these species (Fig. 6, Table 6).

**Table 6:** Statistics for Gene prediction annotation in the *Oryza coarctata* genome.

Method	Software	Species	Gene number
Ab initio	Augustus	—	46,225
	SNAP	—	89,182
Homology-based	GeMoMa	*A. thaliana*	35,284
		*O. brachyantha*	45,135
		*O. punctata*	42,796
		*O. sativa*	48,024
RNAseq	GeneMarkS-T	—	28,041
	PASA	—	35,085
Integration	EVM	—	45,571

### Noncoding RNAs annotation

Non-coding RNA (ncRNA) refers to RNA that does not encode proteins, including various types of functional RNAs such as microRNA, rRNA, and tRNA. Different strategies were used to predict different ncRNAs based on their structural characteristics. The tRNA was identified using tRNAscan-SE v1.3.1^38^. The rRNA prediction was mainly based on the Rfam(v 12.0)^39^ database and predicted using barrnap(v 0.9)^40^. miRNA was identified using the miRBase^41^ database, while snoRNA and snRNA were predicted based on the Rfam(v 12.0) database and using Infenal 1.1^42^. A total of 2,804 tRNAs, 9,075 rRNAs, and 157 miRNAs were predicted (Table 7).

**Table 7:** Statistics for Noncoding RNAs annotation in the *Oryza coarctata* genome.

rRNA number	tRNA number	miRNA number	snRNA number	snoRNA number
9,075	2,804	157	66	331

### Pseudogene prediction

Pseudogenes are sequences similar to functional genes, but they have lost their original function due to mutations such as insertions or deletions. We used GenBlastA v1.0.4^43^ to compare the genome after masking the loci of real genes, in order to identify homologous gene sequences (putative genes). We then used GeneWise v2.4.1^44^ to detect premature stop codons and frameshift mutations in these sequences, and ultimately predicted 28 pseudogenes (Table 8).

**Table 8:** Statistics for Pseudogene prediction in the *Oryza coarctata* genome.

Pseudogene	Stat
Total Number	28
Total length	58,050
Average Length	2,073.21

### Functional annotation of the genome

To annotate the predicted gene sequences, we performed searches against the NR (202009, ftp://ftp.ncbi.nlm.nih.gov/blast/db), EggNOG^45^, GO^46^, SWISS-PROT^47^, and Pfam^48^ databases. Overall, 96.59% of the genes were annotated in these databases (Table 9).

**Table 9:** Statistics for Functional annotation in the *Oryza* coarctata genome.

Database	Annotated Number	Annotated Ratio
GO	34,028	74.67
KEGG	30,669	67.3
KOG	23,113	50.72
Pfam	35,760	78.47
Swissprot	34,057	74.73
TrEMBL	43,983	96.51
eggNOG	36,510	80.11
NR	43,688	95.86
All_Annotated	44,018	96.59

### Discovery of genomic variations among K and L

**Fig. 7:**
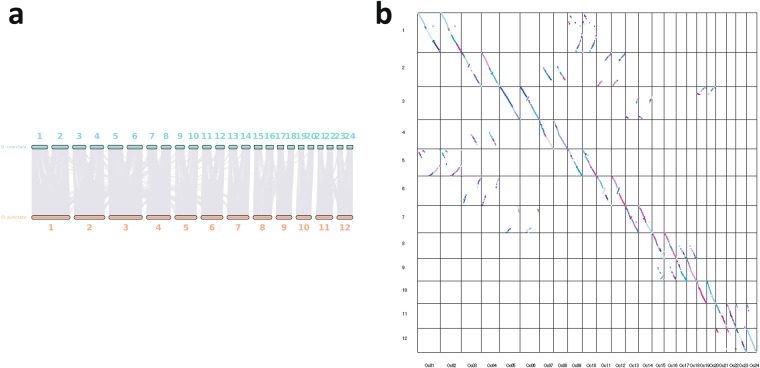
Syntenic blocks between *O. coarctata* and *O. punctata*, represented through a linear collinear graph (**a**) and a dot plot (**b**).

**Fig. 8:**
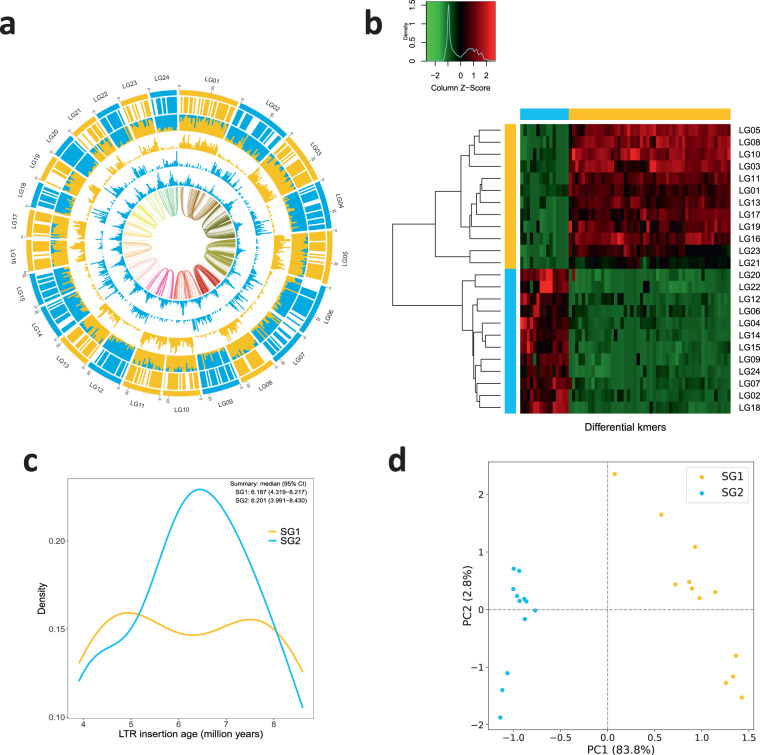
Phased subgenomes of allotetraploid *Oryza coarctata*. (**a**) Chromosomal characteristics (window size: 1 Mb). Rings from outer to inner:(1) Subgenome assignments by a k-Means algorithm. (2) Significant enrichment of subgenome-specific k-mers (blank for non-enriched windows). (3) Normalized proportion of subgenome-specific k-mers. (4–6) Density distribution (count) of each subgenome-specific k-mer set. (7) Density distribution (count) of subgenome-specific LTR-RTs and other LTR-RTs (the most outer, in grey color). (8) Homoeologous blocks of each homoeologous chromosome set. (**b**) Heatmap and clustering of differential k-mers. (**c**) Insertion time of subgenome-specific LTR-RTs. (**d**) Principal component analysis (PCA) of differential k-mers. Points indicate chromosomes.

**Fig. 9:**
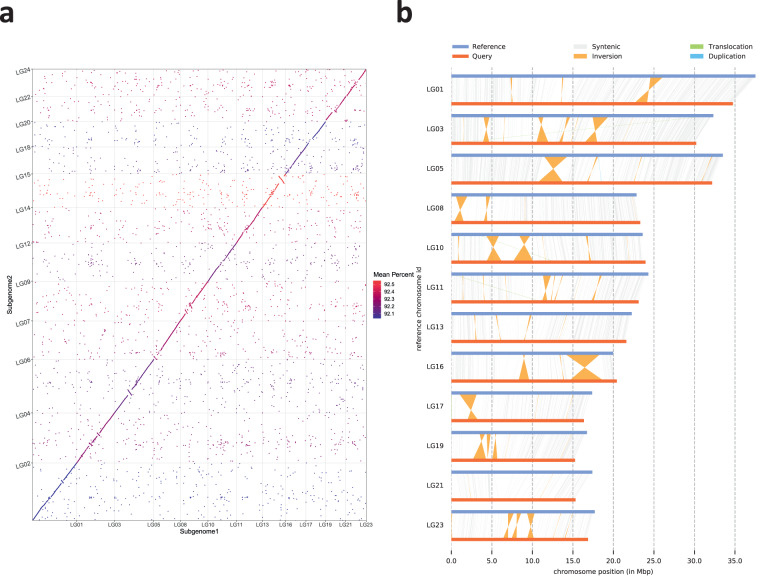
Whole-genome comparison of the Subgenome1 with Subgenome2 assembly. (**a**) Dot plot for the syntenic blocks. (**b**) Chromosome-level local sequence differences.

We utilized the syntenic blocks between *Oryza coarctata* (KKLL) and its related species *Oryza puctata* (BB) (Fig. 7) to uncover a pairing relationship among the 24 chromosomes. Then, using Subphaser^49^ based on the principle of K-mer frequency difference between genomes of different species, we successfully separated the heterozygous chromosomes KK (~271 Mb) and LL (~261 Mb) from the *Oryza coarctata* genome (Fig. 8). A whole-genome synteny analysis was conducted between Subgenome K and Subgenome L using MUMmer, which, as shown in Fig. 9a, revealed a high-level of overall concordance between the K type and L type genomes. To further investigate genomic variations and local differences between the two assemblies, we employed SyRI v1.5 software^50^. This analysis led to the identification of several Mb-sized structural variations such as inversions, translocations, and duplications (Fig. 9b)

## Data Records

The sequencing data, genome assembly and annotation data reported in this paper have been deposited in the Genome Warehouse in National Genomics Data Center (NGDC), Beijing Institute of Genomics, Chinese Academy of Sciences/China National Center for Bioinformation^51^ under the BioProject accession number PRJCA016514 that is publicly accessible at https://ngdc.cncb.ac.cn/gwh. All the clean genome sequencing data including PacBio long-read data^52^, Illumina short-read DNA-seq^53,54^. and Hi-C data^55^, as well as Illumina short-read RNA sequencing data^56^ were deposited in the Genome Sequence Archive (GSA)^57^ of NGDC under the accession number CRA011195. The genome assembly and annotation data have been deposited in the Genome Assembly Sequences and Annotations (GWH) of NGDC under accession number GWHCBHR00000000. The assembled genome has also been deposited in the NCBI assembly with the accession number JAULJY000000000^58^. The annotation results of repeated sequences, gene structure and functional prediction were deposited in the Figshare database^59^.

## Technical Validation

### Assessment of the genome assembly

To evaluate the quality of the assembly, we assessed it from three different perspectives: second-generation data alignment rate, CEGMA evaluation, and BUSCO evaluation. The second-generation data alignment rate was over 99%, indicating the high accuracy of our assembly. Furthermore, the CEGMA evaluation showed that over 98% of the genes and more than 95% of the highly conserved genes were present in the assembly. The BUSCO evaluation also demonstrated the completeness of the assembly, with a score of 97.83% (Tables 10–12).

**Table 10:** Statistics of Second Generation Data Alignment in the *Oryza coarctata* genome.

Total reads	Mapped reads	Mapped (%)	Properly mapped reads	Properly mapped (%)
683,524,014	681,521,913	99.71%	679,605,648	99.43%

**Table 11:** CEGMA assessment results.

Number of 458 CEG* present in assembly	% of 458 CEGs present in assemblies	Number of 248 highly conserved CEGs present	% of 248 highly conserved CEGs present
451	98.47%	236	95.16%

**Table 12:** BUSCO assessment results in the *Oryza coarctata* contig-level genome.

Complete BUSCOs(C)	Complete and single-copy BUSCOs(S)	Complete and duplicated BUSCOs(D)	Fragmented BUSCOs(F)	Missing BUSCOs(M)	Total Lineage BUSCOs
1579 (97.83%)	1111 (68.84%)	468 (29.00%)	7 (0.43%)	28 (1.73%)	1614

Moreover, we evaluated the result of Hi-C based pseudo-chromosomes construction. LACHESIS software was utilized to divide and sequence the genome sequences into groups, while also orienting them. Manual mapping and inspection were then performed to obtain the chromosome level genome version. Our manual checks entailed re-examining the raw Hi-C data, confirming the inconsistency, and determining the correct alignment or orientation based on the highest number of supporting read pairs. Furthermore, the adjustment of placement and orientation errors exhibiting obvious discrete chromatin interaction patterns was performed when the chromatin interaction patterns indicated an arrangement inconsistent with the majority of the data. These adjustments were made based on the same principle of choosing the alignment or orientation that was supported by the highest number of read pairs. After the Hi-C assembly and manual heat map adjustments, it was determined that the 24 chromosomes contained a total of 556,116,023 bp genome sequence, accounting for 96.99% of the sequences located on the chromosomes. Among those sequences located on the chromosomes, the sequence and direction could be determined in 554,379,116 bp, accounting for 99.69% of the total sequence located on the chromosomes.

### Assessment of the genome annotation

**Fig. 10:**
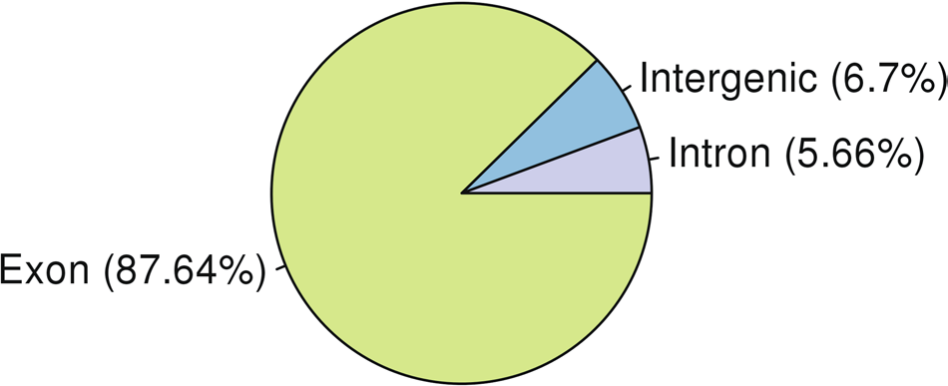
Transcriptome Mapping Statistics.

The number of genes supported by each prediction method was counted, and the majority of the genes were predicted using transcriptome-based and homology-based methods, indicating the high quality of the predictions. The embryophyta database of BUSCO contains 1,614 conserved core genes. We used BUSCO v5.0 software to evaluate the completeness of gene prediction, and 96.22% of BUSCO genes were found in our predicted genes, indicating high completeness (Table 13). The accuracy and completeness of gene prediction were evaluated from the overall level by mapping RNA-seq clean data to the assembled genome using Hisat2 software and calculating and summarizing the coverage of annotated exons, introns, and intergenic regions. In this genome, 87.64% of the transcriptome data mapped to the annotated exons, demonstrating the high accuracy of our prediction model (Fig. 10).

**Table 13:** BUSCO assessment results in the *Oryza coarctata* chromosome-level genome.

Complete BUSCOs(C)	Complete and single-copy BUSCOs(S)	Complete and duplicated BUSCOs(D)	Fragmented BUSCOs(F)	Missing BUSCOs(M)	Total Lineage BUSCOs
1,553 (96.22%)	1,107 (68.59%)	446 (27.63%)	35 (2.17%)	26 (1.61%)	1614

## Data Availability

The versions, settings and options of software tools used in this work are described below: (1) Hifiasm: v0.12, default parameters; (2) CEGMA: v2.5, default parameters; (3) BUSCO: v5, default parameters; (4) HiC-Pro: v2.10.0, default parameters; (5) BWA: 0.7.10-r789, default parameters; (6) LACHESIS, parameters: CLUSTER_MIN_RE_SITES = 78 CLUSTER_MAX_LINK_DENSITY = 2 ORDER_MIN_N_RES_IN_TRUNK = 15 ORDER_MIN_N_RES_IN_SHREDS = 15; (7) Circlize: 0.4.10, default parameters; (8) Diamond v0.9.29.130, default parameters; (9) MCScanX, default parameters; (10) JCVI: v0.9.13, default parameters; (11) VGSC: v2.0, default parameters; (12) RepeatModeler2: v2.0.1, default parameters; (13) RECON: v1.0.8, default parameters; (14) RepeatScout: v1.0.6, default parameters; (15) LTR_retriever: v2.8, default parameters; (16) LTRharvest: v1.5.9, default parameters; (17) LTR_FINDER: v1.1, default parameters; (18) RepeatMasker: v4.1.0, default parameters; (19) MISA: v2.1, default parameters; (20) TRF: v409, parameters:1 1 2 80 5 200 2000 –d -h; (21) Augustus: v2.4, default parameters; (22) SNAP: v2006-07-28, default parameters; (23) GeMoMa: v1.7, default parameters; (24) Hisat: v2.0.4, default parameters; (25) Stringtie: v1.2.3, default parameters; (26) GeneMarkS-T: v5.1, default parameters; (27) Trinity: v2.11, default parameters; (28) PASA: v2.0.2, default parameters; (29) EVM: v1.1.1, default parameters; (30) EggNOG-mapper: v2, default parameters; (31) tRNAscan-SE: v1.3.1, default parameters; (32) Barrnap v0.9, default parameters; (33) Infenal v1.1, default parameters; (34) GenBlastA: v1.0.4, default parameters; (35) GeneWise: v2.4.1, default parameters; (36) InterProScan: v5.34-73.0, default parameters; (37) Subphaser: v1.2, parameters: -q 90; (38) SyRi: v1.5, default parameters; (39) Plotsr: v1.0.0, default parameters; No customized code was developed by the authors.
